# Predictive value of the serum albumin change rate for therapeutic response to targeted therapy in patients with AIDS-related non-Hodgkin lymphoma

**DOI:** 10.3389/fnut.2025.1639609

**Published:** 2025-12-22

**Authors:** Cheng Xingzhen, Yang Jing, Chen Tingyu, Zhao Yong, Wei Guo

**Affiliations:** Department of General Surgery, Chengdu Public Health Clinical Medical Center, Chengdu, Sichuan, China

**Keywords:** Alb change rate, AIDS-related non-Hodgkin lymphoma, targeted therapy, predictive biomarker, nutrition

## Abstract

**Objective:**

This study aimed to evaluate the predictive value of the serum albumin change rate (Alb Change Rate) for treatment efficacy in patients with AIDS-related non-Hodgkin lymphoma (AR-NHL) undergoing targeted therapy (e.g., rituximab), and to explore the clinical implications of serum albumin (Alb) dynamics during treatment.

**Methods:**

This retrospective study included 95 patients diagnosed with AR-NHL between June 2017 and June 2024. The primary endpoint was the therapeutic response after completion of four cycles of treatment regimens containing targeted agents. Patients were categorized into two groups based on treatment response: effective and ineffective. The objective was to investigate the association between the Alb Change Rate and treatment efficacy in AR-NHL patients. Logistic regression analysis was performed to assess the association between the Alb Change Rate and treatment efficacy. Multivariate analysis was used to adjust for potential confounding variables.

**Results:**

Among 95 patients with AR-NHL (mean age: 48.99 ± 12.70 years; 78.95% male). The diffuse large B-cell lymphoma (DLBCL) was the predominant subtype (85.26%). According to Ann Arbor-Cotswolds staging, 75.79% were stage III–IV. After four cycles of targeted therapy, 64 patients (67.37%) responded effectively, while 31 (32.63%) were classified as ineffective, including five deaths. The median Alb Change Rate was 3.09% (−34.71 to 78.55%), with corresponding the Hb-Shift and the CD4^+^Tcell-Shift medians of −5.00 g/L and 10.00 cells/μL, respectively. Common adverse events included gastrointestinal symptoms (92.63%), peripheral neuropathy (92.63%), alopecia (90.53%), pain (43.16%), and bone marrow suppression (32.63%). Univariate analysis showed that Alb Change Rate was significantly associated with treatment response (OR = 116.01; 95% CI: 5.92–2274.51; *p* < 0.01). Patients with Alb Change Rate ≥ 0 had improved outcomes (OR = 4.31; 95% CI: 1.73–10.70; *p* < 0.01). This association remained significant after multivariate adjustment (OR = 9.18; 95% CI: 2.73–30.86; *p* < 0.01).

**Conclusion:**

The Alb Change Rate is a useful predictor of treatment response in AR-NHL patients receiving targeted therapy. Alb Change Rate ≥ 0 was significantly associated with better outcomes. These results highlight the value of dynamic Alb monitoring and nutritional support during treatment. Further prospective studies are needed to confirm these findings.

## Introduction

To date, over 42 million people have died from AIDS-related illnesses, with malignancies remaining a major cause of death in this population (1, 2). Among HIV-associated cancers, AIDS-related lymphoma (ARL) accounts for nearly 40% of cancer-related mortality, with AIDS-related non-Hodgkin lymphoma (AR-NHL)—especially diffuse large B-cell lymphoma (DLBCL)—being the most common subtype (2–4). Due to its subtle onset and HIV-induced immunosuppression, AR-NHL is often diagnosed late and poorly tolerated during treatment, leading to high rates of complications and poor prognosis (2).

Targeted therapies exert their effects by interfering with key signaling pathways involved in tumor cell growth, proliferation, or immune evasion. These therapies include several major classes: monoclonal antibodies (mAbs, e.g., rituximab), small molecule inhibitors (e.g., ibrutinib), antibody-drug conjugates (ADCs, e.g., brentuximab vedotin), bispecific antibodies (BsAbs), immunomodulatory drugs (IMiDs, e.g., lenalidomide), and HDAC/proteasome inhibitors (e.g., bortezomib) (5–8). By targeting specific molecular pathways, these agents reduce off-target toxicity to normal tissues and lower the incidence of complications commonly associated with traditional chemotherapy, such as infections and bone marrow suppression. In addition, targeted therapies can overcome resistance mechanisms and enhance the effectiveness of chemotherapy and radiotherapy, ultimately improving remission rates, quality of life, and long-term survival in patients with AR-NHL (9, 10). However, commonly reported adverse effects of targeted therapies (e.g., diarrhea, nausea, vomiting, and appetite loss) can directly impair nutritional intake and metabolism (11). Emerging evidence suggests that a low body mass index (BMI < 18.5) is an independent predictor of poor prognosis in patients receiving targeted therapy (12).

Nutritional status has long been a critical concern in cancer care. Nutritional interventions aimed at stabilizing body weight have been shown to significantly prolong progression-free survival (PFS) and overall survival (OS) in cancer patients (13). Early identification and correction of nutritional risk in tumor patients have been shown to improve survival outcomes (14, 15). Serum albumin (Alb), a widely used biomarker for nutritional assessment, is often reduced in patients with malnutrition (16). In addition to its role as a nutritional indicator, Alb also binds to and transports various substances such as drugs and hormones, thereby facilitating their distribution throughout the body and enhancing therapeutic efficacy (17). While the role of Alb in oncology has been extensively studied, there remains a lack of research specifically examining its association with treatment response in AR-NHL patients undergoing targeted therapy. Therefore, this study aims to explore the relationship between Alb levels and treatment efficacy in AR-NHL patients receiving targeted agents.

## Patients and methods

### Patients

This retrospective study included 95 patients with AR-NHL who received treatment at the Chengdu Public Health Clinical Medical Center between June 2017 and June 2024. Inclusion Criteria: Confirmed diagnosis of AIDS according to the Chinese Guidelines for Diagnosis and Treatment of HIV/AIDS, based on initial HIV antibody screening and confirmatory testing by the Chinese Center for Disease Control and Prevention; Histopathological and immunohistochemical confirmation of AR-NHL, with tumor staging conducted using the Ann Arbor–Cotswolds classification; Receipt of treatment regimens containing at least one targeted agent (e.g., rituximab, bortezomib); Availability of complete clinical records and Alb data prior to the fifth chemotherapy cycle. Exclusion Criteria: Pregnant or lactating women; Absence of definitive pathological diagnosis; Incomplete clinical data, particularly missing Alb values prior to the fifth chemotherapy cycle; Missing treatment efficacy evaluation data.

### Treatment regimens

All patients received standardized first-line or salvage therapy according to the treatment protocols for AR-NHL. The specific treatment regimens were as follows:

1) R-CHOP regimen: Rituximab 375 mg/m^2^ IV on Day 1, Cyclophosphamide 750 mg/m^2^ IV on Day 1, Doxorubicin 50 mg/m^2^ IV on Day 1, Vincristine 1.4 mg/m^2^ (maximum 2 mg) IV on Day 1, and Prednisone 100 mg orally on Days 1–5. The regimen was repeated every 21 days.
2) R-DA-EPOCH regimen (dose-adjusted): Rituximab 375 mg/m^2^ IV on Day 1, Etoposide 50 mg/m^2^/day by continuous IV infusion on Days 1–4, Doxorubicin 10 mg/m^2^/day by continuous IV infusion on Days 1–4, Vincristine 0.4 mg/m^2^/day by continuous IV infusion on Days 1–4, Cyclophosphamide 750 mg/m^2^ IV on Day 5, and Prednisone 60 mg/m^2^ orally on Days 1–5. The regimen was repeated every 21 days.
3) R-miniCHOP regimen (for elderly or frail patients): Rituximab 375 mg/m^2^ IV on Day 1, Cyclophosphamide 400 mg/m^2^ IV on Day 1, Doxorubicin 25 mg/m^2^ IV on Day 1, Vincristine 1 mg IV on Day 1, and Prednisone 40 mg/m^2^ orally on Days 1–5. The regimen was administered every 21 days.
4) R-HyperCVAD regimen (Part A): Rituximab 375 mg/m^2^ IV on Day 1, Cyclophosphamide 300 mg/m^2^ IV every 12 h for 6 doses (Days 1–3), Vincristine 2 mg IV on Days 4 and 11, Doxorubicin 50 mg/m^2^ IV on Day 4, and Dexamethasone 40 mg orally or IV on Days 1–4 and 11–14. Part A alternated with Part B (methotrexate + cytarabine) every 28 days.
5) R-CVAD regimen: Rituximab 375 mg/m^2^ IV on Day 1, Cyclophosphamide 750 mg/m^2^ IV on Day 1, Vincristine 1.4 mg/m^2^ (maximum 2 mg) IV on Day 1, Doxorubicin 50 mg/m^2^ IV on Day 1, and Dexamethasone 40 mg orally on Days 1–4. The regimen was repeated every 21 days.
6) Bortezomib + EPOCH regimen: Bortezomib 1.3 mg/m^2^ IV on Days 1, 4, 8, and 11; Etoposide 50 mg/m^2^/day by continuous IV infusion on Days 1–4; Doxorubicin 10 mg/m^2^/day by continuous IV infusion on Days 1–4; Vincristine 0.4 mg/m^2^/day by continuous IV infusion on Days 1–4; Cyclophosphamide 750 mg/m^2^ IV on Day 5; and Prednisone 60 mg/m^2^ orally on Days 1–5. The regimen was administered every 21 days.

None of the included patients received concurrent radiotherapy during systemic treatment. Local irradiation was considered only for residual or relapsed lesions after chemotherapy.

### Methods

Demographic and clinical data were collected for all patients, including age, sex, ethnicity, occupation, baseline BMI (BMI-Baseline), and BMI after four cycles of targeted therapy (BMI-After 4 Cycles). Lifestyle factors (smoking, alcohol), comorbidities (hypertension, diabetes, coronary heart disease), and infectious status (HBV, syphilis, tuberculosis, HIV, and ART regimen) were documented. BMI-Shift was calculated as BMI-After 4 Cycles minus BMI-Baseline. Laboratory indices were measured at baseline and prior to the fifth treatment cycle, including white blood cell count, neutrophils, Hb, platelets (PLT), Alb, globulin (Glo), LDH, and CD4^+^/CD8^+^ T-cell counts. Flow cytometry (FC 500, Beckman Coulter) was used for lymphocyte subtyping. Dynamic indices included Hb-Shift (post-treatment Hb minus baseline Hb), Alb Change Rate [(Alb after 4 cycles – Baseline Alb)/Baseline Alb] × 100%, and CD4^+^Tcell-Shift (CD4^+^Tcell-After 4 Cycles minus CD4^+^Tcell-Baseline). Clinical information included lymphoma subtype, initial symptoms, time since onset, Ann Arbor–Cotswolds stage, IPI score, ECOG performance status, PG-SGA score, and nutritional support during treatment. Treatment response was evaluated using RECIST criteria: Complete Response (CR), Partial Response (PR), Stable Disease (SD), and Progressive Disease (PD). Patients were categorized into Effective (CR/PR), Ineffective (SD/PD), and Deceased groups. Adverse events related to treatment were systematically recorded.

### Statistics

Statistical analysis was performed using R4.2.0 and Empower (R) 4.2.0. For normally distributed continuous variables, data were expressed as mean ± standard deviation (Mean ± SD); for non-normally distributed data, the median (M) with interquartile range (IQR) was used. Categorical variables were presented as counts (percentages). Univariate analysis was conducted using multiple linear regression models, *P* than 0.05 is considered statistically significant, and *P* than 0.001 indicates a highly significant statistical difference. Multivariate analysis was performed using generalized linear mixed models, with results expressed as OR (95% Confidence interval (CI)).

## Results

### Clinical characteristics

#### Demographic characteristics

Among 95 patients with AR-NHL, the mean age at diagnosis was 48.99 ± 12.70 years; 75 (78.95%) were male. Most were of Han ethnicity (93.68%), and the remainder from minority groups. Manual laborers comprised 94.74% of the cohort. Smoking and alcohol histories were reported in 29.47 and 17.89% of patients, respectively. Comorbidities included hypertension (6.32%), diabetes (4.21%), coronary artery disease (1.05%), hepatitis B virus (5.26%), syphilis (1.05%), and tuberculosis (1.05%). Six patients (6.32%) were newly diagnosed with HIV at the time of AR-NHL diagnosis; the median duration from HIV diagnosis was 3.00 months (range: 0.00–24.00). Seventeen patients (17.89%) were on integrase strand transfer inhibitor (INSTI)-based ART regimens, while 82.11% received non-INSTI therapies. Twelve (10.43%) had a history of antibody-based therapy. The BMI-Baseline was 22.36 ± 3.48, and BMI-After 4 Cycles was 22.12 ± 3.21. The median BMI-Shift was 0.00 (IQR: −1.23 to 1.00) (Table 1).

**Table 1:** The clinical characteristics 95 cases of AR-NHL.

Characteristics	
Number of cases	95
Age, Mean ± SD, y	48.99 ± 12.70
Sex, *N* (%)	
Man	75 (78.95%)
Women	20 (21.05%)
Ethnicity, *N* (%)	
Han	89 (93.68%)
Tibetan	2 (2.11%)
Yi	2 (2.11%)
Miao	1 (1.05%)
Man	1 (1.05%)
Occupation, *N* (%)	
Physical Labor	90 (94.74%)
Mental Labor	5 (5.26%)
BMI-Baseline, Mean ± SD	22.36 ± 3.48
BMI-After 4 Cycles, Mean ± SD	22.12 ± 3.21
BMI-Shift, M (IQR)	0.00 (−1.23–1.00)
Smoking, *N* (%)	
No	67 (70.53%)
Yes	28 (29.47%)
Alcohol Consumption, *N* (%)	
No	78 (82.11%)
Yes	17 (17.89%)
Hypertension, *N* (%)	
No	89 (93.68%)
Yes	6 (6.32%)
Diabetes Mellitus, *N* (%)	
No	91 (95.79%)
Yes	4 (4.21%)
Coronary Heart Disease, *N* (%)	
No	94 (98.95%)
Yes	1 (1.05%)
HBV	
No	90 (94.74%)
Yes	5 (5.26%)
Syphilis, *N* (%)	
No	94 (98.95%)
Yes	1 (1.05%)
Tuberculosis, *N* (%)	
No	94 (98.95%)
Yes	1 (1.05%)
Duration since confirmed HIV infection, *M* (IQR), months	3.00 (0.00–24.00)
Initial diagnosis of HIV infection, *N* (%)	
No	6 (6.32%)
Yes	89 (93.68%)
ART Regimens, *N* (%)	
INSTIs	17 (17.89%)
Non-INSTIs	78 (82.11%)
Treated with ABT, *N* (%)	
No	84 (88.42%)
Yes	12 (10.43%)

#### Laboratory characteristics

We compared laboratory indicators measured at initial diagnosis and prior to the fifth cycle of targeted therapy to evaluate dynamic changes during treatment. The Hb-Shift was −5.00 g/L (range: −19.50 to 5.00), and the median Alb change rate (Alb Change Rate) was 3.09% (range: −34.71 to 78.55%). Based on Alb Change Rate, patients were stratified into two groups: Decrease group (Alb Change Rate < 0): 39 patients (41.05%), Stable or increase group (Alb Change Rate ≥ 0): 56 patients (58.95%) (Table 2).

**Table 2:** The laboratory characteristics of AR-NHL in 95 patients.

Laboratory tests	Baseline	After 4 cycles
	Mean ± SD or *M* (IQR)	Mean ± SD or *M* (IQR)
WBC, 10^9/L	5.51 ± 1.85	3.84 (2.63–4.79)
Neu, 10^9/L	3.27 (2.55–4.56)	2.20 (1.29–2.88)
Hb, g/L	125.08 ± 20.25	117.68 ± 22.89
PLT, 10^9/L	203.53 (84.90)	226.00 (155.00–295.00)
Alb, g/L	37.45 ± 6.25	39.00 ± 6.32
Glo, g/L	32.64 ± 7.65	26.33 ± 5.10
A/G ratio	1.21 ± 0.34	1.54 ± 0.40
LDH, U/L	276.00 (208.00–584.50)	256.50 ± 107.52
CD4^+^Tcell, cells/μl	220.00 (144.50–329.00)	207.00 (146.00–386.50)
CD8^+^Tcell, cells/μl	472.00 (347.50–645.00)	399.00 (264.50–470.00)
CD4^+^Tcell/CD8 + Tcell ratio	0.42 (0.25–0.72)	0.53 (0.35–0.82)
Hb-Shift, g/L	−5.00 (−19.50–5.00)	
2Alb change rate, %	3.09 (−34.71–78.55)	
Alb change rate group, *N* (%)		
<0	39 (41.05)	
> = 0	56 (58.95)	
CD4^+^Tcell-shift, cells/μl	10.00 (−100.00–87.50)	

#### Tumor and treatment characteristics

Of the 95 AR-NHL patients, 81 (85.26%) had DLBCL, followed by Burkitt lymphoma (BL, 9.47%), plasmablastic lymphoma (PCL, 3.16%), and single cases of marginal zone lymphoma (MZL) and follicular lymphoma (FL). Initial symptoms included lymphadenopathy (74.47%), abdominal pain (14.89%), painful nodes (6.38%), and fever (8.60%), with a median diagnostic delay of 2.00 months (range: 1.00–3.00). According to Ann Arbor–Cotswolds staging: Stage I (4.21%), II (21.05%), III (30.53%), and IV (44.21%). Based on Ann Arbor-Cotswolds classification: Type A (54.74%); Type B (45.26%). International Prognostic Index (IPI) scores ranged from 0 to 5, with 44.68% scoring ≥3. ECOG PS was ≥2 in 42 patients (45.26%). Nutritional risk (PG-SGA) at diagnosis was high (Grade C or D) in 70 patients (73.68%). During treatment, 47 patients (49.47%) received nutritional support (Table 3).

**Table 3:** The basic profile of AR-NHL in 95 patients.

Tumor overview	
Pathological diagnosis, *N* (%)	
DLBCL	81 (85.26%)
BL	9 (9.47%)
PCL	3 (3.16%)
MZL	1 (1.05%)
FL	1 (1.05%)
Symptoms, *N* (%)	
Lymphadenopathy	70 (74.47%)
Axilla	31 (32.98%)
Neck	16 (17.02%)
Head and face	13 (13.83%)
Inguinal	7 (7.45%)
Lower limbs	2 (2.13%)
Supraclavicular region	1 (1.06%)
Buttocks	1 (1.06%)
Abdomen	1 (1.06%)
Multifocal throughout the body	1 (1.06%)
Lymph node pain	6 (6.38%)
Abdominal pain	14 (14.89%)
Cough	8 (8.60%)
Abdominal distension	7 (7.45%)
Foreign body sensation in throat	5 (5.32%)
Limb swelling	4 (4.26%)
Lumbar pain	4 (4.26%)
Fever	4 (4.26%)
Chest tightness	2 (2.13%)
Shortness of breath	1 (1.06%)
Headache	1 (1.06%)
Jaw pain	1 (1.06%)
Chest pain	1 (1.06%)
Hip joint pain	1 (1.06%)
Poor appetite	1 (1.06%)
Nosebleed	1 (1.06%)
Disease duration, *M* (IQR), *M*	2.00 (1.00–3.00)
Ann Arbor-Cotswolds staging, *N* (%)	
I	4 (4.21%)
II	20 (21.05%)
III	29 (30.53%)
IV	42 (44.21%)
Ann Arbor-Cotswolds classification, *N* (%)	
A	52 (54.74%)
B	43 (45.26%)
IPI Score, *N* (%)	
0	10 (10.64%)
1	15 (15.96%)
2	23 (24.47%)
3	23 (24.47%)
4	19 (20.21%)
5	4 (4.26%)
ECOG PS, *N* (%)	
0	30 (31.58%)
1	23 (24.21%)
2	31 (32.63%)
3	8 (8.42%)
4	3 (3.16%)
5	0 (0.00%)
PG. SGA, *N* (%)	
A	4 (4.21%)
B	21 (22.11%)
C	28 (29.47%)
D	42 (44.21%)
Nutritional support, *N* (%)	
No	48 (50.53%)
Yes	47 (49.47%)

Treatment response (per RECIST) showed CR in 4.21%, PR in 63.16%, SD in 17.89%, and PD in 14.74%. Overall, 64 patients (67.37%) had an effective response (CR + PR), and 31 (32.63%) were ineffective (SD + PD). There were 5 deaths (5.26%) during treatment (Table 4). Common adverse events included gastrointestinal symptoms (92.63%), peripheral neuropathy (92.63%), alopecia (90.53%), pain (43.16%), and bone marrow suppression (32.63%). Other events included liver dysfunction (27.37%), fever (21.05%), and sepsis (5.26%). Eight patients (8.42%) required chemotherapy modification due to severe intolerance (Table 5).

**Table 4:** Treatment response of AR-NHL in 95 patients.

Treatment response and efficacy evaluation	
RECIST, *N* (%)	
CR	4 (4.21%)
PR	60 (63.16%)
SD	17 (17.89%)
PD	14 (14.74%)
Treatment efficacy, *N* (%)	
Effective	64 (67.37%)
Ineffective	31 (32.63%)
Death toll, *N* (%)	5 (5.26%)

**Table 5:** Chemotherapy complications of AR-NHL in 95 patients.

Chemotherapy complications	*N (%)*
Gastrointestinal Reactions	88 (92.63%)
Peripheral Neuropathy	88 (92.63%)
Hair Loss	86 (90.53%)
Pain	41 (43.16%)
Bone Marrow Suppression	31 (32.63%)
Liver Dysfunction	26 (27.37%)
Fever	20 (21.05%)
Sepsis in AIDS	5 (5.26%)
Pneumonia	2 (2.11%)
Renal Dysfunction	1 (1.05%)
Tumor Lysis Syndrome	1 (1.05%)
Acute Heart Failure	1 (1.05%)
Chemotherapy Regimen Change	8(8.42%)

### Correlation between Alb change rate and treatment efficacy

#### Univariate analysis of treatment response

Univariate logistic regression identified a significant association between the Alb Change Rate and treatment efficacy (OR = 116.01; 95% CI: 5.92–2274.51; *p* < 0.01). To further investigate this relationship, patients were stratified based on the Alb Change Rate. Stratified analysis showed that patients with Alb Change Rate ≥ 0 had a significantly higher likelihood of treatment response compared to those with a decrease (OR = 4.31; 95% CI: 1.73–10.70; *p* < 0.01). These findings suggest that a post-treatment increase in Alb may indicate better therapeutic outcomes (Table 6).

**Table 6:** Univariate analysis for treatment efficacy.

Covariate	Statistics	OR (95%CI)	*p*-value
Sex			
Man	75 (78.95%)	Reference	
Women	20 (21.05%)	0.66 (0.24, 1.84)	0.43
Age	48.99 ± 12.70	1.01 (0.97, 1.04)	0.69
BMI-Baseline	22.36 ± 3.48	1.16 (1.01, 1.34)	0.04
BMI-Shift	0.00 (−1.23–1.00)	0.92 (0.77, 1.12)	0.42
Duration since confirmed HIV infection	3.00 (0.00–24.00)	0.99 (0.98, 1.01)	0.45
ART Regimens, *N* (%)			
INSTIs	17 (17.89%)	Reference	1.00
Non-INSTIs	78 (82.11%)	1.57 (0.54, 4.63)	0.41
Hb	125.08 ± 20.25	1.00 (0.98, 1.03)	0.70
Hb-Shift	−5.00 (−19.50–5.00)	0.99 (0.97, 1.01)	0.36
Alb	37.45 ± 6.25	1.03 (0.96, 1.11)	0.37
Alb Change Rate	3.09% (−34.71–78.55%)	116.01 (5.92, 2274.51)	<0.01
Alb change rate group			
<0	39 (41.05%)	Reference	
> = 0	56 (58.95%)	4.31 (1.73, 10.70)	<0.01
CD4^+^Tcell, cells/ul	220.00 (144.50–329.00)	1.00 (1.00, 1.00)	0.80
CD4^+^Tcell-shift, cells/ul	10.00 (−100.00–87.50)	1.00 (1.00, 1.00)	0.17
Ann Arbor-Cotswolds staging			
I, II	24 (25.26%)	Reference	
III, IV	71 (74.74%)	0.46 (0.15, 1.36)	0.16
IPI score			
0	10 (10.64%)	Reference	
1	15 (15.96%)	1.62 (0.19, 13.93)	0.66
2	23 (24.47%)	0.47 (0.08, 2.76)	0.40
3	23 (24.47%)	0.33 (0.06, 1.88)	0.21
4	19 (20.21%)	0.34 (0.06, 2.07)	0.24
5	4 (4.26%)	0.75 (0.05, 11.65)	0.84
ECOG PS			
0–1	53 (55.79%)	Reference	
> = 2	42 (44.21%)	0.29 (0.12, 0.71)	0.01
PG. SGA	7 (3–13)	0.95 (0.88, 1.02)	0.15
Nutritional support, *N* (%)			
No	48 (50.53%)	Reference	
Yes	47 (49.47%)	0.88 (0.37, 2.08)	0.77

#### Multivariate analysis of Alb change rate and treatment efficacy

**Figure 1:**
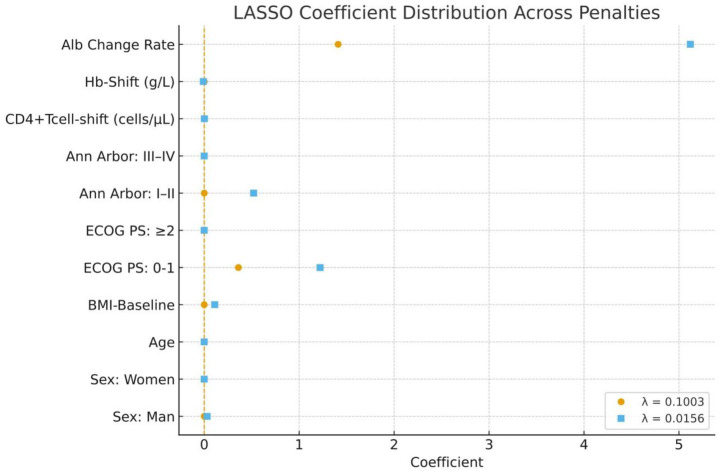
Forest plot of LASSO regression coefficients. The coefficients of variables change under two different penalty parameters (*λ* = 0.1003 and *λ* = 0.0156). Variables with coefficients farther from zero exert a stronger influence on treatment efficacy prediction.

Before conducting multivariate logistic regression analysis, we first applied Least Absolute Shrinkage and Selection Operator (LASSO) regression for variable regularization. At a relatively small regularization parameter (*λ* = 0.0156), the following variables were identified as having potential influence on the model: male sex, BMI-Baseline, ECOG PS, Ann Arbor–Cotswolds staging, CD4^+^Tcell-Shift, Hb-Shift, and Alb Change Rate (Table 7 and Figure 1). Based on these, stepwise logistic models were constructed. The Alb Change Rate remained significantly associated with treatment efficacy in the fully adjusted model (OR = 678.70; 95% CI: 20.19–22820.04; *p* < 0.05), which accounted for age, sex, BMI-Baseline, ECOG PS, Ann Arbor stage, CD4^+^Tcell-Shift, and Hb-Shift. Further stratification showed that patients with Alb Change Rate ≥ 0 had markedly higher odds of treatment response compared to those with a decline, even after adjustment (OR = 9.18; 95% CI: 2.73–30.86; *p* < 0.01) (Table 8). These results support Alb Change Rate as an independent predictor of therapeutic efficacy in AR-NHL.

**Table 7:** LASSO regression analysis of variables associated with treatment efficacy.

Variable	Coefficient (*λ* = 0.1003)	Coefficient (*λ* = 0.0156)
(Intercept)	0.45	−2.73
Sex		
Man	0.00	0.03
Women	0.00	≈0.00
Age	0.00	0.00
BMI-Baseline	0.00	0.11
ECOG PS		
0–1	0.36	1.22
> = 2	≈0.00	≈0.00
Ann Arbor-Cotswolds staging		
I, II	0.00	0.52
III, IV	0.00	0.00
CD4^+^Tcell-shift, cells/ul	0.00	0.002
2Hb-Shift	0.00	−0.01
3Alb Change Rate	1.41	5.12

**Table 8:** Relationship between the Alb change rate and treatment efficacy.

Outcome	Crude model		Model I		Model II	
	OR (95%CI)	*P*-value	OR (95%CI)	*P*-value	OR (95%CI)	*P*-value
Alb change rate	116.01 (5.92, 2274.51)	0.0017	111.53 (5.63, 2208.93)	0.0020	678.70 (20.19, 22820.04)	0.0003
Alb change rate group						
<0	Reference		Reference		Reference	
> = 0	4.31 (1.73, 10.70)	0.0017	4.21 (1.68, 10.56)	0.0022	9.18 (2.73, 30.86)	0.0003

### Representative imaging findings

**Figure 2:**
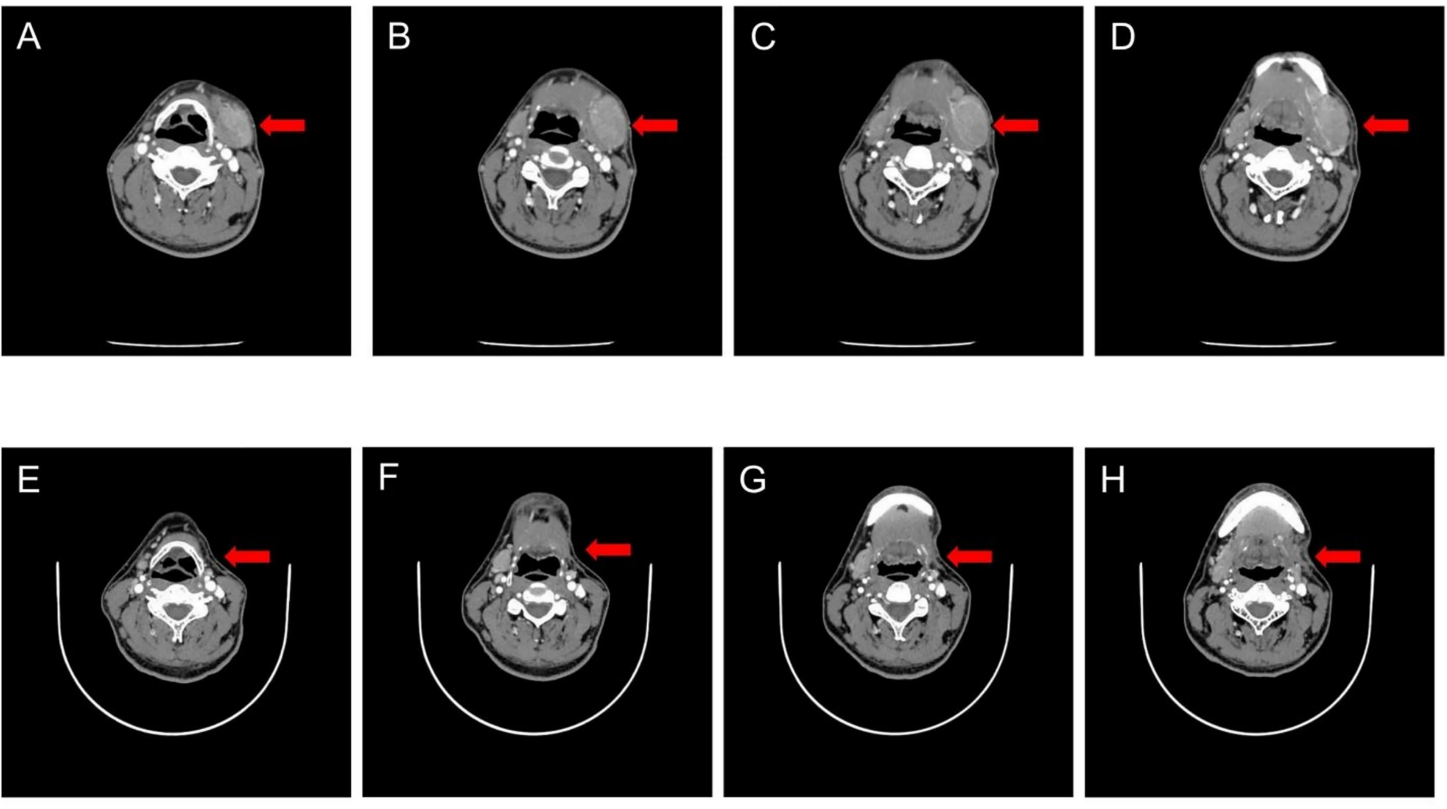
Representative contrast-enhanced CT images of a patient with AIDS-related non-Hodgkin lymphoma (AR-NHL) before and after four cycles of R-CHOP chemotherapy. Panels **(A–D)** represent contrast-enhanced CT images of the patient’s neck obtained at initial diagnosis; panels **(E–H)** show follow-up contrast-enhanced CT images of the neck acquired after 4 weeks of treatment. The left submandibular lymphoma of the patient showed a marked regression after treatment.

To visually demonstrate tumor response, we selected a representative patient with AR-NHL who was initially admitted to our hospital with progressive enlargement of the left submandibular lymph node for more than 3 months. The patient received four regular cycles of the R-CHOP chemotherapy regimen starting on December 19, 2021. Post-treatment contrast-enhanced computed tomography (CT) imaging showed resolution of the left submandibular lymphoma lesion (Figure 2), consistent with the patient’s clinical improvement and a positive Alb Change Rate (68.20%). This imaging finding further supports the predictive association between dynamic changes in serum albumin and treatment efficacy.

## Discussion

This single-center retrospective study included 95 AR-NHL patients. Painless lymphadenopathy was the most common initial symptom, and most cases lacked specific clinical features. At diagnosis, the majority were in intermediate or advanced stages, aligning with prior findings on ARL (3). Although 93.68% had initiated ART, the median duration was short (3 months), and baseline CD4^+^ T cell counts were low (median 220.00 cells/μL), indicating poor immune function. Given the high proportion of manual laborers, delayed HIV diagnosis and ART initiation may reflect socioeconomic and educational barriers, which could contribute to inadequate immune recovery and subsequent AR-NHL development (2, 18, 19). Nutritional indicators such as BMI and Alb are established prognostic factors in HIV-associated lymphomas (20). In this cohort, nutritional status was generally poor—only 4.21% were well-nourished (PG-SGA category A)—highlighting the need for nutritional support. Malnutrition may result from immune dysregulation, tumor microenvironment alterations, and chronic inflammation (21).

Alb, a liver-synthesized protein, plays crucial roles in maintaining oncotic pressure, transporting molecules, buffering pH, and acting as an antioxidant and nutritional biomarker (17). Numerous studies have demonstrated Alb’s prognostic significance in both malignancies and HIV/AIDS-related conditions. In advanced non-small cell lung cancer (NSCLC), higher baseline Alb and minimal early decline were linked to improved OS during immune checkpoint inhibitor therapy (22). Similarly, in gastric cancer, Alb and composite indices such as the combined Alb–neutrophil-to-lymphocyte ratio (COA–NLR) independently predicted OS (23, 24). In head and neck squamous cell carcinoma (HNSCC), pretreatment Alb levels were associated with better disease-free and overall survival (25). Among hospitalized AIDS patients, higher admission Alb correlated with reduced 12-week mortality (26). In ARL, hypoalbuminemia was significantly associated with poorer OS, and Alb was confirmed as an independent prognostic marker (27).

With the growing body of research on Alb in oncology, an increasing number of studies have demonstrated a strong association between Alb levels and the efficacy of targeted therapies. In a retrospective study, Fiala et al. (28) analyzed 457 patients with advanced NSCLC receiving erlotinib treatment and found that pretreatment Alb levels were independent predictors of both progression-free survival (PFS) and overall survival (OS). Similarly, another study reported that in NSCLC patients undergoing targeted therapy, both baseline and week-12 Alb levels were significantly associated with OS. Furthermore, the study showed that dynamic changes in Alb levels during treatment could serve as prognostic indicators, highlighting Alb as a valuable biomarker for evaluating the prognosis of patients receiving targeted therapy (29). Our study also supports the association between Alb levels and the efficacy of targeted treatment. Specifically, in patients with AR-NHL, an increase in Alb following targeted therapy (Alb Change Rate ≥ 0) was indicative of a favorable treatment response.

To further investigate the relationship between changes in albumin (Alb) levels and the efficacy of targeted therapy in patients with AIDS-related non-Hodgkin lymphoma (AR-NHL), both before and after treatment, we innovatively introduced the Alb Change Rate as a novel indicator. This parameter, reflecting Alb variation relative to baseline, showed a significant association with treatment efficacy (OR = 116.01; 95% CI: 5.92–2274.51; *p* < 0.01). Patients with the Alb Change Rate ≥ 0 had a markedly higher response rate than those with declining levels (OR = 4.31; 95% CI: 1.73–10.70; *p* < 0.01). After adjusting for age, sex, BMI, Ann Arbor stage, ECOG PS, CD4^+^T cell shift, and Hb shift, the association remained significant (OR = 9.18; 95% CI: 2.73–30.86; *p* < 0.01), indicating that Alb Change Rate may serve as an independent predictor of therapeutic response in AR-NHL.

Alb may influence the efficacy of targeted therapies in cancer patients through multiple mechanisms, including nutritional status, inflammatory responses, drug metabolism, and immune function. In patients with advanced NSCLC, those with Alb levels above 35 g/L derived significantly greater benefit from targeted therapies, while those with lower Alb levels experienced limited therapeutic effects (29). Malnutrition itself can impair T cell function by altering metabolic pathways, thereby compromising the effectiveness of immune responses (30). In addition, Alb is considered a negative acute-phase reactant. Inflammatory cytokines such as IL-1, IL-6, and TNF-*α* can suppress hepatic Alb synthesis and promote its degradation, resulting in decreased Alb levels (31). The ratio of Alb to CRP, referred to as the CRP-to-Alb ratio (CAR), has been proposed as a predictive marker for targeted therapy efficacy, with higher CAR values associated with poorer prognosis (32). Alb also functions as a primary drug-binding protein in plasma. Many targeted agents—including sorafenib and erlotinib—exhibit high Alb-binding affinity (33, 34). For common targeted therapies used in AR-NHL, IgG-based monoclonal antibodies (e.g., rituximab) and Alb are recycled through the neonatal Fc receptor (FcRn) pathway. FcRn binds both IgG and Alb in acidic endosomal environments, protecting them from lysosomal degradation and prolonging their half-life. Inflammatory conditions or immune dysfunction may disrupt this recycling process, resulting in reduced Alb levels, which could in turn affect antibody pharmacokinetics and therapeutic efficacy (35, 36). Bispecific antibodies (BsAbs), which also utilize the IgG backbone, share this FcRn–HSA transport mechanism to extend their half-life. Engineering approaches that fuse Alb-binding domains (ABDs) or Alb itself into BsAbs have been shown to enhance tumor targeting and molecular stability (37). Antibody-drug conjugates (ADCs), composed of IgG antibodies linked to cytotoxic payloads, have similarly incorporated HSA to improve *in vivo* stability and tumor delivery efficiency (38). As the most abundant plasma protein, HSA serves as the principal carrier for many small-molecule drugs. Agents such as ibrutinib and bortezomib exhibit high Alb-binding affinity. When Alb levels are reduced, drug-binding sites are limited, increasing the proportion of free (unbound) drug in circulation. While this may enhance therapeutic potency, it also elevates the risk of adverse effects and premature treatment discontinuation (39). Alb levels have also been used to predict drug tolerability and toxicity (40, 41). Although immunomodulatory drugs (IMiDs) primarily cross cell membranes via non-saturable passive diffusion and exhibit relatively low protein-binding affinity (38), hypoalbuminemia—as an indicator of systemic inflammation or malnutrition—has been shown to correlate with reduced IMiD efficacy (42).

This study has several limitations. As a single-center retrospective analysis with a relatively small sample size, the findings lack external validation and may not be generalizable. The study population was limited to Chinese patients, introducing potential regional and ethnic bias, and excluded pregnant and lactating women. Therefore, larger multicenter prospective studies are needed to confirm the predictive value of the Alb Change Rate and assess the impact of nutritional interventions on Alb dynamics, particularly in the context of widespread ART use and the unique clinical features of AR-NHL in China.

In conclusion, we recommend dynamic monitoring of Alb levels throughout the course of targeted therapy in patients with AIDS-related non-Hodgkin lymphoma (AR-NHL). For patients exhibiting a decline in Alb, timely nutritional support or early intervention should be considered to improve treatment outcomes and enhance the effectiveness of immunotherapy.

## Conclusion

This study demonstrates that most patients with AIDS-related non-Hodgkin lymphoma (AR-NHL) present at an intermediate or advanced stage at their initial diagnosis. Alb Change Rate was identified as an independent indicator for evaluating the efficacy of targeted therapy in AR-NHL patients. An increase in Alb after treatment (Alb Change Rate ≥ 0) was associated with favorable therapeutic outcomes, suggesting its potential as a predictive biomarker. We recommend close monitoring of dynamic changes in Alb during targeted therapy for AR-NHL and timely nutritional or clinical interventions when Alb levels decline. Given the inherent limitations of retrospective analysis, further prospective studies are warranted to validate the predictive value of Alb Change Rate for treatment response.

## Data Availability

The original contributions presented in the study are included in the article/supplementary material, further inquiries can be directed to the corresponding author.
